# Using behavioural design and theories of change to integrate communication solutions into health systems in India: evolution, evidence and learnings from practice

**DOI:** 10.1136/ihj-2022-000139

**Published:** 2022-12-29

**Authors:** Priyanka Dutt, Anna Godfrey, Sara Chamberlain, Radharani Mitra

**Affiliations:** 1 BBC Media Action India, New Delhi, India; 2 BBC Media Action, London, UK

**Keywords:** Communication, Health behavior, Information technology, Consumer health information

## Abstract

Between 2011 and 2019, an integrated communication programme to address reproductive, maternal, neonatal and child health was implemented in the Indian state of Bihar. Along with mass media, community events and listening groups, four mobile health services were co-designed with the government of Bihar. These were *Mobile Academy*—a training course for frontline health workers (FLHWs) supporting them as the last mile of the health system; *Mobile Kunji*—a job aid to support FLHWs’ interactions with families; *Kilkari*—a maternal messaging service delivering information directly to families’ mobile phones, encouraging families to seek public health services through their FLHWs; and *GupShup Potli*—mobile audio stimulus used by FLHWs in community events. While *Mobile Kunji* and *GupShup Potli* scaled to other states (two and one, respectively), neither was adopted nationally. The Government of India adopted *Kilkari* and *Mobile Academy* and scaled to 12 additional states by 2019. In this article, we describe the programme’s overarching person-centred theory of change, reflect on how the mHealth services supported integration with the health system and discuss implications for the role of health communication solutions in supporting families to navigate healthcare systems. Evaluations of *Kunji, Academy* and *GupShup Potli* were conducted in Bihar between 2013 and 2017. Between 2018-2020, an independent evaluation was conducted involving a randomised controlled trial for *Kilkari* in Madhya Pradesh; qualitative research on *Kilkari* and *Academy* and secondary analyses of call record data. While the findings from these evaluations are described elsewhere, this article collates key findings for all the services and offers implications for the role digital and non-digital communication solutions can play in supporting joined-up healthcare and improving health outcomes.

**WHAT IS ALREADY KNOWN ABOUT THIS SUBJECT**

- Direct to consumer (D2C) mobile communication programmes offer great potential to disseminate health information direct to families, rapidly, at scale and at low cost. Similarly mobile health (mHealth) tools designed to support client–provider interaction have potential for improving the reach and quality of health information and advice at the last mile in low and middle-income countries. Yet, little evidence exists on how these two different approaches can be designed in a complementary manner to reach and impact different audiences and help families navigate healthcare systems.

**WHAT DOES THIS STUDY ADD**

- This article reviews the design and impact of four mobile health services and how they support health system integration. Originally conceived as a complementary suite of mobile services developed under a single digital theory of change nested under a broader programme theory of change, this article outlines how each service was designed and subsequently scaled. While the findings from evaluations of these services are described elsewhere, this article reviews the key impact of the four services against the original theory of change, and offers implications for the role digital and non-digital communication solutions can play in supporting joined-up healthcare and improving health outcomes.

**HOW MIGHT THIS IMPACT ON CLINICAL PRACTICE OR FUTURE DEVELOPMENTS**

- Review findings highlight that D2C mobile communication programmes and mHealth tools designed to support FLHW communication with families can support healthcare integration by increasing reach and supporting person-centred healthcare. By enabling FLHWs and families to build on their own capabilities, they also improve health outcomes. Yet these two different approaches reach and impact different audiences, highlighting that D2C digital communication services need to be complemented by sustained face-to-face communication by trained, well equipped FLHWs to address deep-seated behaviours under normative influence.

## Introduction

Between 2011 and 2019, BBC Media Action and its partners implemented the Shaping Demand and Practices (SDP) project to improve family health in Bihar. Delivered in collaboration with the state government and funded by the Bill & Melinda Gates Foundation, SDP aimed to tackle the high maternal and child mortality rates in Bihar, using several different forms of communication to extend high-quality health information and advice to rural populations. SDP was part of the wider Ananya (Hindi for "unique") programme to improve reproductive, maternal, neonatal and child health (RMNCH) by addressing supply-side and demand-side barriers to adoption, coverage, quality, equity and health impact.^1^ To complement these wider effects to improve outreach of FLHWs, SDP had a particular focus on improving the quality of engagement between FLHWs and families by refreshing FLHW knowledge of RMNCH behaviours and improving communication between the health system (FLHW) and families. While communication outputs were rooted in Bihar, there was an intention to see the project as a ‘laboratory’’ where approaches could be tested for national scale up.

While several communication outputs from the project were scaled beyond Bihar by the government, this article focuses on digital services, including: (1) *Mobile Academy*, designed to refresh FLHW’s knowledge and improve their communication skills, (2) *Mobile Kunji* (guide), an audio-visual job aid for FLHWs, combining audio content with a printed deck of cards, to use during counselling sessions with families, (3) *GupShup Potli* (bagful of chatter), short, dramatised ‘audio-capsules’ for families, designed to spark discussion facilitated by FLHW at monthly village health events and (4) *Kilkari* (baby’s gurgle), which sought to improve families’ knowledge of healthy RMNCH practices and increase integration between communities and the health system by both reaching families directly and reinforcing the information communicated by FLHWs. All four services used interactive voice response (IVR) technology, providing audio content to anyone with even the most basic phone.

## Pathway to practice using human-centred and gender intentional design

SDP’s theory of change was informed by a range of social, behavioural and communication theories that provided a framework to identify determinants of health behaviour.^2–5^ These included factors such as knowledge, attitudes, self-efficacy (people’s confidence in their ability to practice a behaviour) and discussion. Also important were social norms, health service access and quality and socioeconomic factors. The project was shaped by the hypothesis that encouraging “simple, doable actions” (such as saving money for transport to hospital) would build confidence and, over time, encourage people to take up behaviours that may appear more difficult to achieve initially (such as using modern contraceptives).^6^

**Figure 1:**
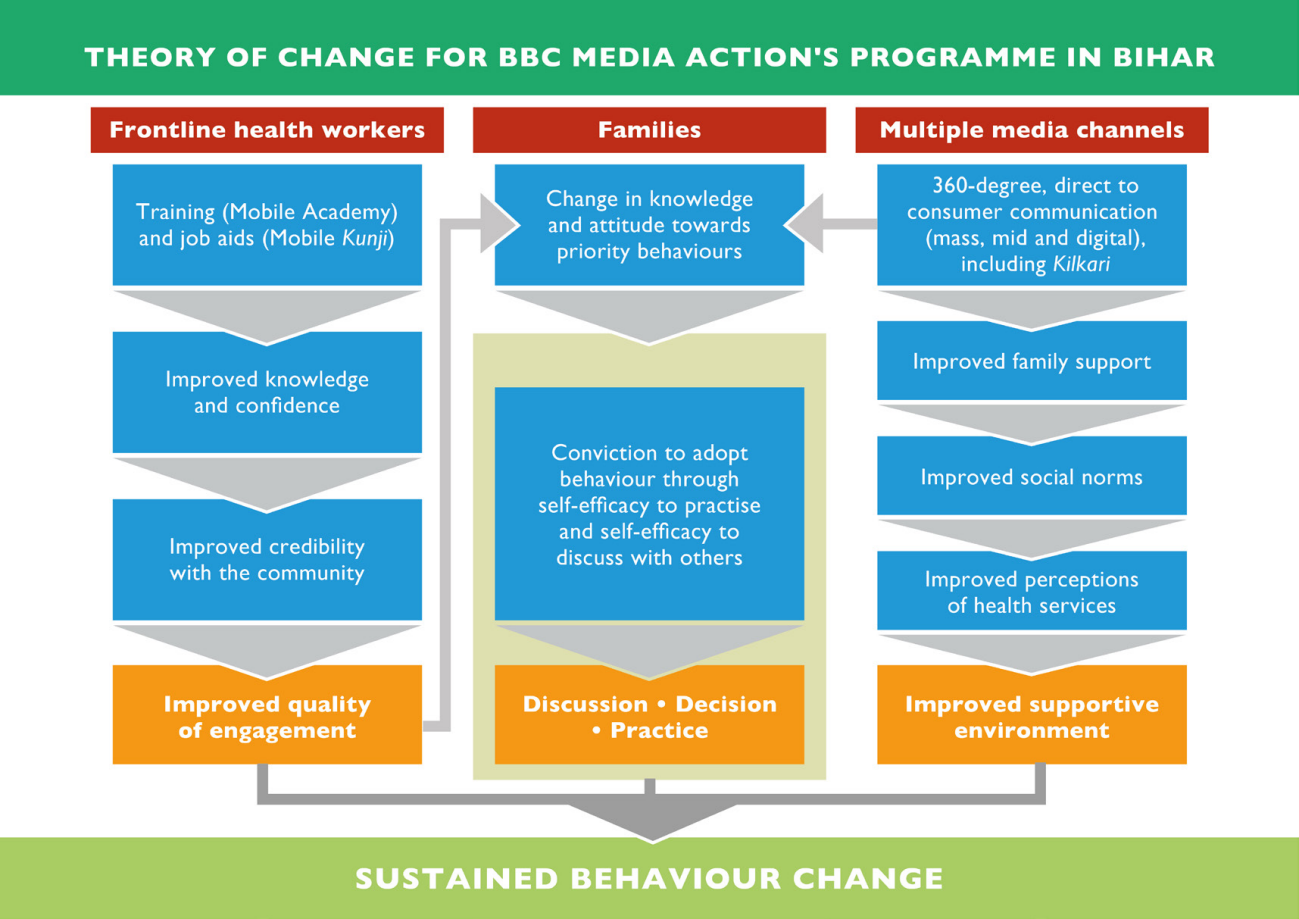
The Shaping Demand and Practices theory of change.

To stimulate demand for health services and strengthen key health behaviours, SDP focused on creating more connections between the community and the health system and for those connections to be more engaging. It recognised the importance of FLHW motivation in their retention and performance^7^ as well as achieving improvement in health outcomes and universal health coverage.^8^ FLHWs needed support to become better motivated and respected, and more effective training to refresh their knowledge and help them persuade families to adopt healthy practices. The project sought to go ‘beyond health’ to focus on professional pride, credibility, agency and empowerment to engage and motivate FLHWs in Bihar. We hypothesised that an integrated communication intervention would affect changes in health outcomes by: (1) increasing knowledge of, and changing attitudes towards, RMNCH practices and services, (2) improving the quality of family–FLHW engagement through improving FLHW’s knowledge, confidence and their credibility with families, (3) triggering more discussion about health practices, both within the family and between the family and FLHW, (4) increasing adoption of positive RMNCH behaviours and (5) contributing to a more supportive environment for changes in practice (figure 1). With the gender digital divide in India, all the digital services needed to be informed by gender-intentional design and research driven processes and were underpinned by a five-stage User-Centred Design process^9^. They aimed to contribute to the Government of India’s commitment to universal basic healthcare and overcome some of the social and cultural barriers that impede FLHW–family interactions such as lack of respect or trust.

## Combining lived experience with strategy

Formative research showed that more than half the women in Bihar were illiterate, with little access to mass media. But Bihar’s FLHWs, coupled with high levels of household phone ownership, presented an opportunity.^10^ A deeper dive into mobile phone usage among FLHWs and families showed that digital literacy levels were incredibly low requiring insightful and strategic service and content design to be effective.^11^

**Figure 2:**
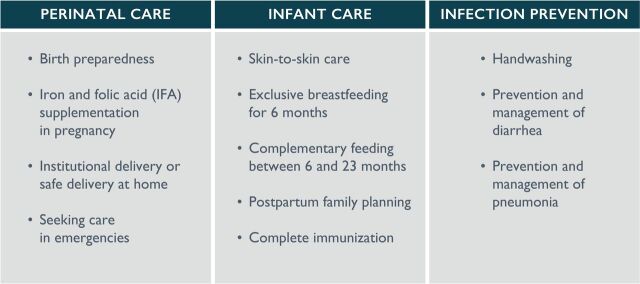
Priority RMNCH behaviours.

This research informed the development of a detailed communication strategy to generate demand for health services and strengthen the practice of 11 ‘priority RMNCH behaviours’, which spanned a 1000-day window from conception until the baby is two years old (figure 2).

### Identifying influencers of behaviour

Behaviours were distinguished by the factors that influenced them. For instance, the high rate of institutional deliveries was believed to be influenced by government incentives. Immediate breast feeding was almost entirely dependent on health practitioners at institutions. When the benefit of practicing a specific behaviour was not immediate, obvious or visible, encouraging change was much more challenging. Additionally, behaviours such as exclusive breast feeding and complementary feeding were influenced by social norms, habits and rituals in the community. Mothers-in-law and the wider community had a significant influence on these behaviours, while the public health system and health practitioners had less involvement.

### Creating surround sound

**Figure 3:**
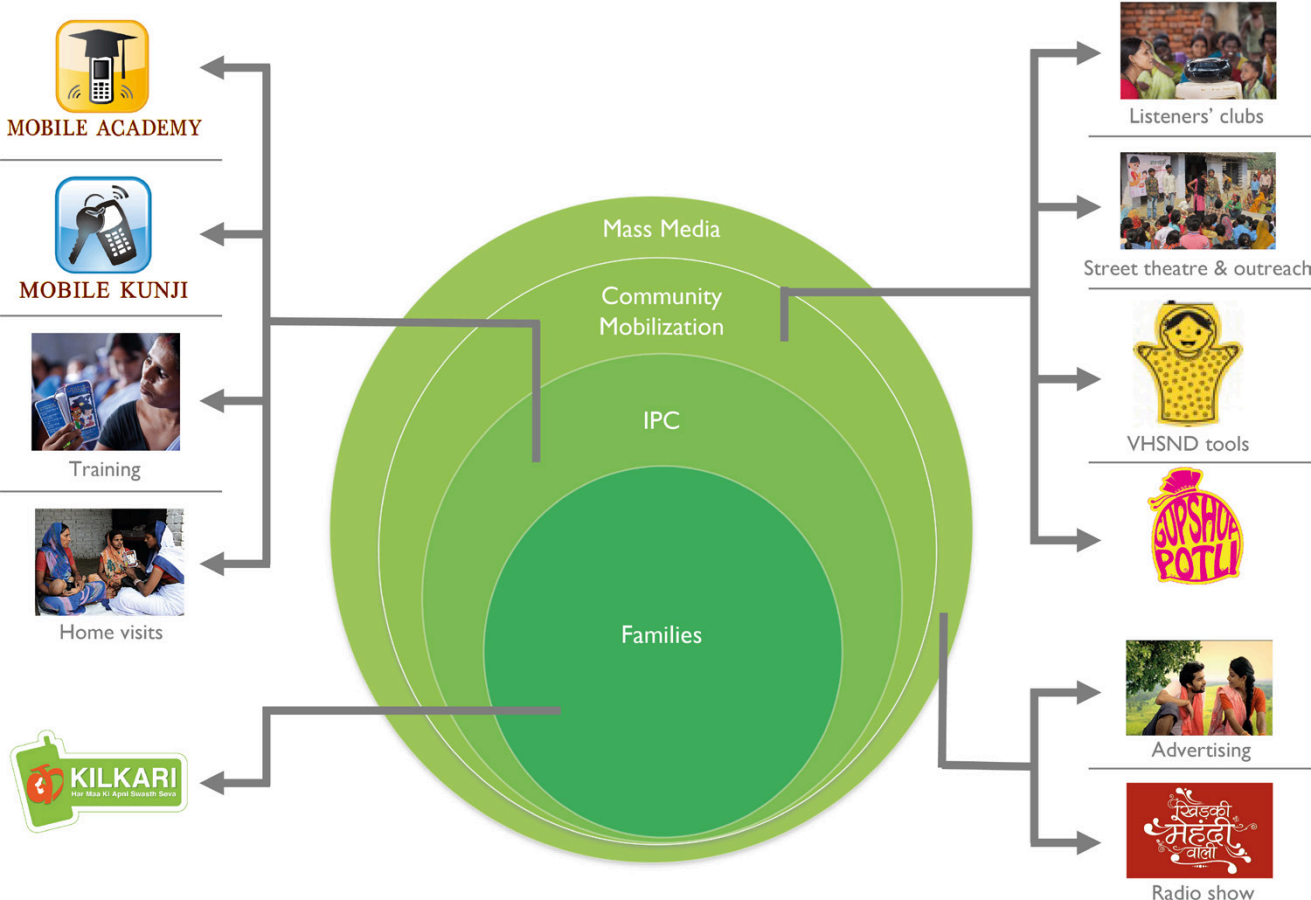
The media mix in Bihar. IPC refers to interpersonal communication. VHSND refers to the village health, sanitation and nutrition day events held in villages across the state each month.

The project adopted an integrated layered approach, where communication via different channels reinforced each other, to enable repeated exposure to new ideas and to create a sense of normality around new behaviours (figure 3). It emphasised ‘surrounding’ target audiences with multiple yet complementary channels of communication, recognising that different types of communication elicit different psychological responses. A layered communication approach is believed to improve diffusion of information, increase credibility and provide a greater chance of exposure to content.

### Digital solutions for social and behaviour change

**Figure 4:**
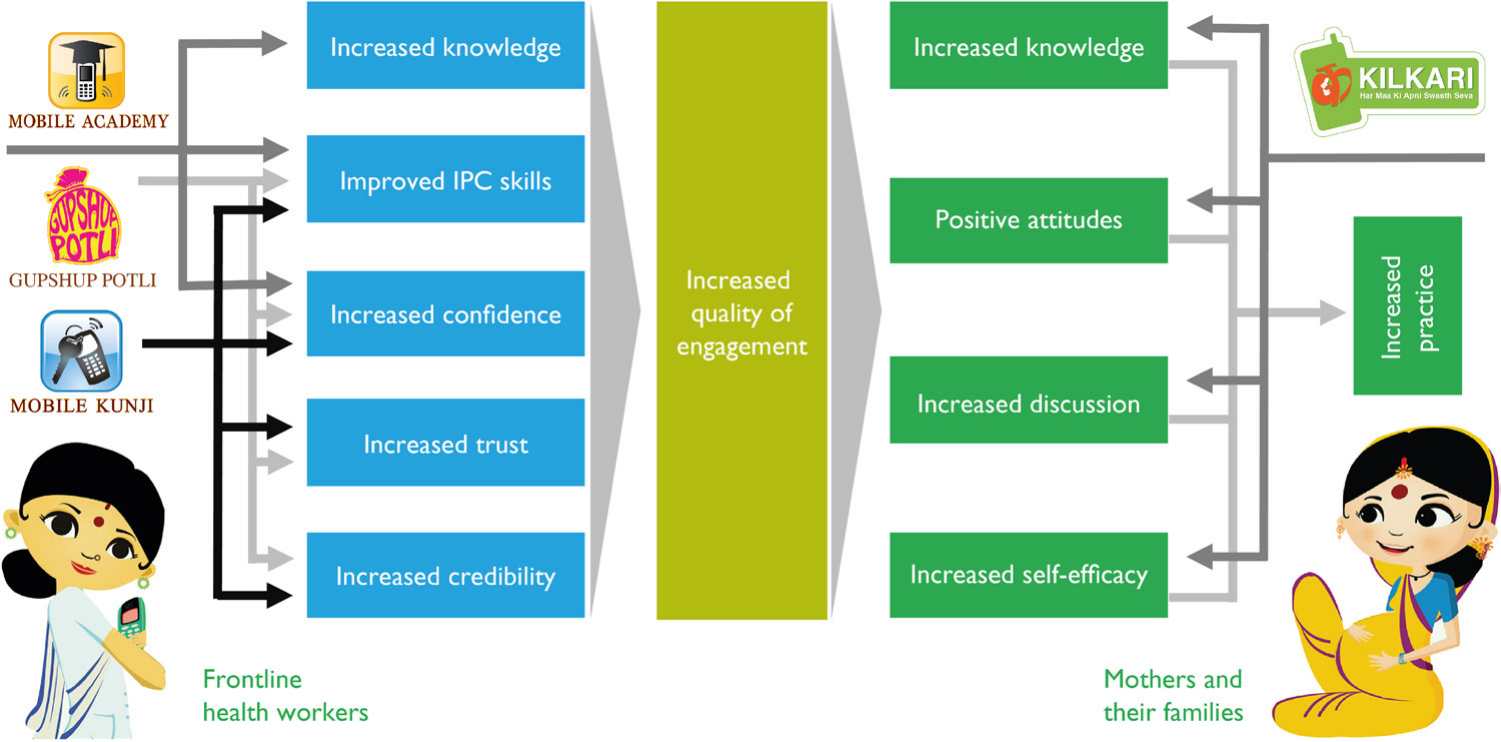
Digital theory of change. IPC refers to interpersonal communication.

Nested in the broader programmatic theory of change, a complementary suite of mHealth services was developed under a single digital theory of change (figure 4), based on the premise that (1) providing timely, accessible, accurate and relevant information improves knowledge, (2) while knowledge is necessary, it is insufficient to change behaviour, (3) negotiations between FLHWs and families are critical to improving the self-efficacy of families, (4) discussion is often a gateway to change and (5) increased knowledge and better interpersonal communication skills will make FLHWs more confident and motivated to better support families to navigate the health system and make the healthiest possible choices.

### Direct-to-consumer versus facilitated communication

While *Mobile Academy* and *Kilkari* were delivered directly to consumer (eg, FLHWs and families, respectively), *Mobile Kunji* and *GupShup Potli* were designed to support facilitated communication between FLHWs and their clients. *Mobile Academy* delivered training on negotiated communication to FLHWs and was complemented by *Mobile Kunji*, a job aid designed to support FLHWs’ communication with families. *GupShup Potli* content used open-ended questions to encourage discussion among larger groups of listeners who met monthly at village health events. All three services were intended to incrementally improve the relationship between families and the health system, at the very last mile of service delivery. *Kilkar*i was designed to reinforce what families heard from their FLHWs or reach those people—particularly marginalised groups—underserved by FLHWs. All four services were intended to complement each other, layering communication with families from different sources—that is, directly through *Kilkari*, in personalised conversations with their FLHWs, and in group discussions with other families. They supported families with the knowledge and skills to find and use RMNCH services in the healthcare system, for example, navigational competence.

The content for each of the services was devised to address a different part of the digital theory of change. FLHWs accessed *Mobile Academy* by dialling a number and worked their way through chapters, lessons and quizzes, learning as they went. Each lesson was divided into three sections—the top provided the FLHW with tips and tricks on how best to convince a family to adopt new practices; the middle demonstrated how she could present information to the family and the end was a rhyming couplet recapping the key information for the lesson.

The middle and end of each *Mobile Academy* lesson was replicated on *Mobile Kunji*. However, *Kunji* needed to be used flexibly, allowing FLHWs to respond to the specific information needs of the different women they met in the day. For example, an FLHW scheduled to meet a pregnant woman as well as one with a five-month-old baby would need to be able to communicate about birth preparedness, family planning, nutrition and childhood illnesses, all in one day. *Kunji*, therefore, broke up complex health information into simple, doable actions, delivered through a printed deck of cards and mobile-based audio content. The FLHW and family together could choose what specific information to focus on. Choice and agency in terms of information-seeking behaviour were driven by discussions between the FLHW and the family. The content spanned a range of different determinants of behaviour from the theory of change: increasing knowledge, shifting attitudes, addressing norms, driving discussion and modelling practice.

*GupShup Potli*, on the other hand, focused on a single health topic each month rather than tailoring discussion to individual users’ information needs. It was designed to encourage people at different life stages to participate in common discussions—helping shape how the entire community thought or felt about a specific behaviour. FLHWs were able to choose from a limited menu of content, to shape the discussions they wanted to focus on that month. Content modelled various attitudes, norms and conversation triggers that provided a stimulus for communities and FLHWs to explore issues together.

*Kilkari* offered the most tailored communication based on the woman’s stage of pregnancy or childcare, designed to generate demand for health services and products, and echo what communities heard from FLHWs. The delivery of content had to be timed so that the right information reached the right recipient at the right time. Multiple rounds of user-testing demonstrated that *Kilkari* needed to be the simplest of all four services, because it was designed to be delivered directly to rural users with minimal access to information, and would not be facilitated. The content in *Kilkari* was simplified to focus on improving knowledge, providing timely reminders, encouraging information-seeking, promoting discussion and encouraging healthy practices. It did not address any of the normative issues underlying these health behaviours.

All four services were delivered in the voice of a doctor character—Dr Anita.^9^ In a state with a low doctor to patient ratio, the choice of a doctor character was a deliberate one, designed to bring an unfamiliar and distant health system closer to families. The use of Dr Anita’s ‘voice’ across services added to the layering of communication and helped reinforce information accessed in different contexts and conversations.

### Digital alone cannot build health equity

The theory of change was underpinned by the principle of fair and just access to health information and healthcare irrespective of income, religion, caste, class, creed, sex or location. It recognised that digital alone would struggle to build health equity and there was a need to use different pathways to reach people. Therefore, the theory of change recognised that facilitated communication by FLHWs trained and furnished with job aids, along with mass media and community outreach would be required to reach the poorest and most marginalised.

Even the most accessible digital channel (IVR) would still exclude families without phones. Despite this limitation, *Kilkari* was designed to reach the poorest and most marginalised mobile phone owning households.^9^ By delivering high-quality, accessible content every week, *Kilkari* aimed to supplement the face-to-face interactions that families had with FLHWs, while encouraging them to speak directly to FLHWs for more information. It also offered the opportunity to access more sensitive information—such as family planning—confidentially.

*Mobile Kunji* and *GupShup Potli* were not constrained by mobile phone access in the household. Instead, there were dependent on FLHW’s phone access, digital literacy and interactions with families. Equipped with *Kunji,* FLHWs would visit a family between 12 and 15 times in the 1000-day period. Families would listen to *GupShup Potli* at the monthly village events they attended to access public health services. However, while this facilitated communication was essential to building health equity, the theory of change recognised that it was constrained by the number of interactions that FLHWs achieved with families.

## Partnering with government for scale

Priorities for public funds are extensive and complex, making it necessary and appropriate for governments to make hard choices about what is scaled. Several SDP outputs, including advertising, radio, school activities, street theatre and the mobile services described here, were adopted and scaled by government in other states or nationally. But they were all scaled independently, rarely overlapping and, therefore, not layered in the way SDP was designed.

However, government adoption and integration with national health databases enabled *Kilkari* and *Mobile Academy* to be made available for all relevant users registered in those databases in 13 states. By April 2019, when BBC Media Action transitioned *Kilkari* and *Academy* to the government and their local partner, Armman, over 206,000 FLHWs had completed *Mobile Academy* and *Kilkari* had reached 10 million subscribers, demonstrating unprecedented scale.^12^ According to 2022 statistics (available on the Armman website at the time of writing), *Kilkari* has reached a total of 24.6 million subscribers across 17 states and *Academy* over 235,000 FLHWs across 16 states.

## Impact on health outcomes and integration

An independent qualitative assessment of *Mobile Academy* found that the service bolstered FLHWs’ RMNCH knowledge and provided them with positive ways to communicate with families.^13^ An independent analysis of system-generated call record data found that over four in five FLHWs (81%) who started *Mobile Academy* completed the IVR training, and only 1% did not ‘pass’ (i.e. accurately answer at least half the quiz questions)^14^ suggesting the service was effective.

Based on an individually randomised control trial (RCT) in Madhya Pradesh published elsewhere, *Kilkari* had a significant effect on a limited number of behaviours, namely modern contraceptive use (driven largely by condom use) and child immunisation at 10 weeks. Effects on family planning were substantially larger for those exposed to half or more of the relevant calls. Also importantly, the analysis found greater effects for more marginalised families exposed to *Kilkari* (e.g. poorer subscribers and those from disavdantaged castes exposed to family planning calls were more likely to adopt modern contraceptives, and less educated families exposed to immunisation calls were more likely to immunise their baby at 10 weeks).^15^ Using the RCT intervention data, an equity analysis found the poorest subscribers, educated to at least a secondary level, appear to have had the biggest equity gains from exposure to *Kilkari*.^16^

While family planning and immunisation were the two main topics covered in the 72 calls (18% and 13% respectively), *Kilkari* did not, however, have an impact on any other health practices covered in the calls, including infant and young child feeding practices— entrenched behaviours that are under normative influence and have little engagement with health systems.^17^ Contrary to the theory of change, *Kilkari* did not change women’s reported discussion or demand for information across all the behaviours.^15^

In contrast, an independent review of *Mobile Kunji* evaluation data in Bihar found that exposure to *Kunji* was associated with longer interactions with FLHWs and women discussing the information more with others-often with their family.^18^ Women exposed to Mobile Kunji were more trusting of their FLHWs and more likely to agree with the information provided by their FLHW, compared with those who were unexposed.^18^ FLHWs credited Mobile Kunji for building their confidence and knowledge, and for improving clients’ trust, comprehension and acceptance of the information they were providing.^19^ Unlike *Kilkari*, *Mobile Kunji* was associated with improved knowledge and practices across a number of RMNCH behaviours commonly under normative influence such as: birth preparedness, iron folic acid consumption, exclusive breastfeeding, likelihood to have initiated complementary feeding by six months of age and implementing appropriate dietary diversity.^18^

The evaluation of *GupShup Potli* was the weakest in design—based on cross-sectional data—with multiple risks of bias. Despite these limitations, exposure to *GupShup Potli* was associated with improved health-related knowledge, greater interpersonal discussion and higher reported rates of iron and folic acid consumption and current use of contraception.^20^ Evidence suggests that the four digital solutions not only improved health outcomes, but also supported key principles of integration including a focus on people (eg, patient focus) and optimising reach (eg, maximising coverage),^21^ by enabling FLHWs and families to build on their own capabilities.

## Conclusion

The SDP theory of change and evaluations of *Mobile Academy*, *Mobile Kunji* and *GupShup Potli* demonstrate the importance of supporting FLHWs as change agents by improving their interpersonal communication skills, thereby contributing to behaviour change. As the last mile of health system service delivery, the quality of interactions with FLHWs can often determine whether families feel they can trust and navigate the health system. The evaluation of *Kilkari* demonstrates that digital D2C communication solutions, when strategically designed with inclusion, gender and equity in mind, can change a limited number of health behaviours—particularly among poorer, less educated or more marginalised groups. The digital facilitated communication tools (ie, *Mobile Kunji* and *GupShup Potli* that support FLHW communication) were associated with improvements in FLHW performance as well as generating interpersonal discussion unlike *Kilkari*—the digital D2C service. Deep-seated normative behaviours where families have little engagement with the health system—like complementary feeding —saw more positive associations when facilitated communication tools such as *Mobile Kunji* —were used. This highlights the role of digital solutions in supporting people-centred care and improving FLHW’s performance.

While the impact of the layered “surround sound” communication approach in Bihar was not studied, the individual evaluations of the digital services suggest the value of layering communication and engaging people in multiple ways to support joined up care and improve health outcomes. Sustained integrated social and behaviour change communication (SBCC), using multiple media touchpoints and formats, including digital tools, is likely required to shift social norms and change deeply entrenched behaviours. These could be complemented by narrative entertainment which has proven effective at shifting social norms.^22–24^ These conclusions build on recommendations by Scott K et al that digital D2C communication needs to be complemented by sustained face-to-face communication by trained, well equipped FLHWs to address entrenched behaviours under normative influence.^17^ In addition, gender intentional design and equity analysis in SBCC strategies are critical to ensure that marginalised women without access to phones are not left behind.^9 25^
