# Correction: Using behavioural design and theories of change to integrate communication solutions into health systems in India: eEvolution, evidence and learnings from practice

**DOI:** 10.1136/ihj-2022-000139corr1

**Published:** 2023-02-21

Dutt P, Godfrey A, Chamberlain S, *et al*. Using behavioural design and theories of change to integrate communication solutions into health systems in India: evolution, evidence and learnings from practice. *Integ Health J* 2022;4:e000139. doi: 10.1136/ihj-2022-000139

This article has been corrected since it was first published online. The 'What Does This Study Add' and Conclusion paragraphs have been updated.

